# Exercise-based rehabilitation reduces reinjury following acute lateral ankle sprain: A systematic review update with meta-analysis

**DOI:** 10.1371/journal.pone.0262023

**Published:** 2022-02-08

**Authors:** Jente Wagemans, Chris Bleakley, Jan Taeymans, Alexander Philipp Schurz, Kevin Kuppens, Heiner Baur, Dirk Vissers

**Affiliations:** 1 Department of Rehabilitation Sciences and Physiotherapy, Faculty of Medicine and Health Sciences, University of Antwerp, Antwerp, Belgium; 2 Department of Health Professions, Bern University of Applied Sciences, Bern, Switzerland; 3 School of Health Science, Ulster University, Newtownabbey, Northern Ireland; 4 Faculty of Physical Education and Physiotherapy, Vrije Universiteit Brussel, Brussels, Belgium; Goethe University Frankfurt: Goethe-Universitat Frankfurt am Main, GERMANY

## Abstract

**Research questions:**

1) Do exercise-based rehabilitation programs reduce re-injury following acute ankle sprain?; 2) Is rehabilitation effectiveness moderated by the exercise’s therapeutic quality, content and volume?

**Methods:**

This systematic review with meta-analysis (PROSPERO: CRD42020210858) included randomized controlled trials in which adults who sustained an acute ankle sprain received exercise-based rehabilitation as an intervention. Databases CINAHL, Web of Science, SPORTDiscus, Cochrane Central Register of Controlled Trials, PEDro and Google Scholar were searched for eligible articles (last search: March 2021). ROB II screening tool by Cochrane was used to assess risk of bias and the i-CONTENT tool was used to assess quality of interventions. Both qualitative analysis and quantitative data synthesis were performed.

**Results:**

Fourteen randomized controlled trials comprising 2182 participants were included. Five studies were judged overall low risk of bias and i-CONTENT assessment showed poor to moderate therapeutic quality of exercise across all included articles. Pooled data found significant reductions in re-injury prevalence at 12 months, in favour of the exercise-based rehabilitation group vs usual care (OR: 0.60; 95%CI: 0.36 to 0.99). Pooled data for re-injury incidence showed not-significant results (MD: 0.027; 95%CI: -2.14 to 2.19). Meta-regression displayed no statistically significant association between training volume and odds of re-injury (r = -0.00086; SD: 0.00057; 95%CI: -0.00197 to 0.00025). Results from patient-reported outcomes and clinical outcomes were inconclusive at 1 month, 3–6 months and 7–12 months of follow up.

**Conclusion:**

Exercise-based rehabilitation reduces the risk of recurrent ankle sprain compared to usual care, but there is insufficient data to determine the optimal content of exercise-based interventions. Training volume varied considerably across studies but did not affect the odds of sustaining a re-injury. Effects on patient-reported outcomes and clinical outcomes are equivocal. Future research should compare different exercise contents, training volumes and intensities after ankle sprain.

## Introduction

Ankle sprains are amongst the most common musculoskeletal injuries in sports [1]. Doherty et al. [2] pooled data from prospective studies, reporting a cumulative incidence rate of 11.5 ankle sprains/1000 exposures and a prevalence of 11.8%. Lateral ankle sprains (LAS) are the most common [2–4], and usually involve excessive inversion-internal rotation either with or without plantar flexion. The Anterior Talofibular Ligament (ATFL) [5] has the lowest load tolerance [5] and is most frequently injured [1, 5–10]. In more severe ankle sprains the Calcaneofibular Ligament (CFL) [5, 6, 8, 10], Anterior Inferior Tibiofibular Ligament (AITFL) [6, 8], Posterior Inferior Tibiofibular Ligament (PITFL) [5, 6, 8] or even the Deltoid Ligament [6] are involved.

The average time to return to sport after lateral ankle sprain is 16 to 24 days [5, 11–15], but a large proportion of athletes experience re-injury or other long-term problems [1, 4, 16–21]. Epidemiology data of recurrent ankle sprains in athletes range from 12% to 47%, with the largest re-injury rates occurring in junior basketball (47%), volleyball (46%) and American Football (43%) [22]. Chronic ankle instability (CAI) is characterised by recurrent ankle sprains, feelings of the ankle “giving away” and perceived instability [4, 23–25]. It is estimated that up to 40% of people develop CAI, which may also be an important mediator for post traumatic osteoarthritis [4]. Ankle sprains incur a substantial economic impact [26], mostly because of the substantial indirect costs due to decreased physical activity, diminished productivity and lower quality of life [4].

A previous systematic review based on studies published up to 2017 [7], included seven randomized controlled trials (RCTs) examining the effectiveness of rehabilitation exercises after an acute ankle sprain. Their pooled data found significant reductions in re-injury risk over a 12 month period with exercise based rehabilitation, but a sensitivity analysis also indicated that effect magnitudes were reduced in higher quality studies. The authors were also unable to make any clear recommendation on the optimal content and volume of the exercise-based intervention programs, due to insufficient reporting. Total rehabilitation time has been shown to be a key moderator of treatment effect in other musculoskeletal research; for example, the risk of anterior cruciate ligament (ACL) injury is optimally reduced with higher training doses (>0.75 hours/week) and more than 14 months of follow-up [27].

It is important to update existing evidence, as new studies emerge and new methods develop. Since January 2017, many new studies have been published in this field. The most recent systematic review in this field focused primarily on re-injury outcomes. Recent guidelines informing clinical assessment and return to play decisions after ankle sprain [8, 28] highlight the importance of quantifying both mechanical and sensorimotor impairments, using a combination of clinical and patient reported outcomes. The ankle sprain literature has also not yet been examined using the international Consensus on Therapeutic Exercise aNd Training tool (i-CONTENT tool), which provides an objective evaluation of the therapeutic quality of ankle sprain rehabilitation programs, and the potential impact on treatment effect estimates [29]. The aim of this review is to update the evidence base informing the management of acute ankle sprains, by including newly emerged studies, performing a more comprehensive review of clinical and patient reported outcomes, objectively assessing quality of interventions, and by including quantitative analysis to examine the effect of training volume on re-injury. The specific research questions are: 1) Does exercise-based rehabilitation reduce reinjury following acute ankle sprain?; 2) Is rehabilitation effectiveness moderated by the exercise’s therapeutic quality, content, and volume?

## Methods

### Selection and search strategy

The protocol was registered a priori with the International Prospective Registration Register of Systematic Reviews (PROSPERO) on November 24^th^, 2020 (registration number: CRD42020210858, https://www.crd.york.ac.uk/prospero/display_record.php?ID=CRD42020210858). The preferred Reporting Items for Systematic Reviews and Meta-Analyses (Prisma) guidelines were followed, and depicted in S3 and S4 Files [30]. The MEDLINE search strategy is shown in S1 File. This search strategy was modified as required and applied across multiple databases (CINAHL, Web of Science, SPORTDiscus, Cochrane Central Register of Controlled Trials, Physiotherapy Evidence Database (PEDro) and Google Scholar). All databases were searched from their inception through March 2021. Furthermore, additional literature was hand-searched, by going over reference lists of screened articles. To be included in this study, articles had to meet the following predetermined eligibility criteria (Box 1). Eligibility screening was done by two independent reviewers (JW & AS) in two phases: first screening of titles and abstracts, then reading full texts. The Rayyan app was used for both screening phases [31]. Studies which were excluded in the second phase of screening are referenced in S2 File. In case of disagreement a third reviewer (CB) was invited to reach consensus.

**Box 1. Eligibility criteria**

$$\begin{array}{ll} \text{Study}\;\text{design} & \text{Randomized}\;\text{controlled}\;\text{trials}\\ \text{Participants} & \text{Patients}\;\text{with}\;\text{acute}\;\text{ankle}\;\text{sprain}\\ \text{Intervention} & \text{Exercise-based}\;\text{rehabilitation}\;\text{strategies},\;\text{in}\;\text{isolation}\;\text{or}\;\text{as}\;\text{an}\;\text{adjunct}\;\text{to}\;\text{usual}\;\text{care}\\ \text{Outcome}\;\text{measures} & \text{Re-injury}\\ & \text{Pain}\\ & \text{Patient-reported}\;\text{outcome}\;\text{measures}\\ & \text{Clinical}\;\text{outcomes}\\ \text{Comparison} & \text{Exercise-based}\;\text{rehabilitation}\;\text{compared}\;\text{to}\;\text{usual}\;\text{care}\\ & \text{Usual}\;\text{care}\;\text{plus}\;\text{exercise-based}\;\text{rehabilitation}\;\text{compared}\;\text{to}\;\text{usual}\;\text{care}\\ & \text{Different}\;\text{types}\;\text{of}\;\text{exercise-based}\;\text{rehabilitation}\;\text{compared}\;\text{to}\;\text{each}\;\text{other} \end{array}$$

### Data extraction

Data extraction was also undertaken independently by two reviewers (JW & AS) as per the published protocol. Extraction of data comprised methodological data (authors, year of publication, language, study design, participants demographics, inclusion/exclusion criteria, recruitment site and time, time since injury at recruitment, diagnosis, ankle injury history, time of follow-up, content of exercise-based intervention and comparison intervention) and details of exercise interventions (duration of the intervention, number of sessions, time per rehabilitation session). We also calculated the total rehabilitation time to estimate training volume. This calculation was based on duration of one rehabilitation session (minutes), duration of the entire intervention program (weeks) and number of rehabilitation sessions included in the intervention program. Any disparities regarding extracted data were resolved by consensus discussion with the third reviewer (CB).

### Risk of bias

Risk of bias was independently evaluated (JW & AS) using the Risk of Bias in randomized controlled trials (ROB II) [32]. This is screening tool which is based on five categories: 1) randomisation process; 2) deviation from the intended intervention; 3) missing outcome data; 4) measurement of the outcome; 5) selection of the reported results. Each domain comprises relevant questions and a judgement for each respective domain via an algorithm that considers the answers to the relevant questions to propose a final judgement [32]. Consensus was reached by consulting the third reviewer (CB), in case of disparities.

### Quality of intervention

The international Consensus on Therapeutic Exercise aNd Training (i-CONTENT) tool, published in 2020 [29], assesses seven items: patient selection, dosage of the exercise programme, type of exercise programme, qualified supervisor, type and timing of outcome assessment, safety of the exercise programme and adherence to the exercise programme [29]. All items were independently evaluated (JW & AS) as either “low risk” or “high risk” of ineffectiveness of the exercise intervention. In case of equivocality, the item could also be evaluated as “probably done” or “probably not done”. Each evaluation should be substantiated by a rationale to support the evaluation.

### Data analysis

Meta-analysis was performed on re-injury data by one data analyst (JT). All Meta-analyses and forest plots were conducted and established using Comprehensive Meta-analysis software (CMA 2^nd^ version- Biostat, Englewood, USA). If a study reported no re-injuries, the value “1” was added to both groups to be able to calculate Odds Ratio’s (OR). When studies only reported graphical data, values were estimated using the WebPlotDigitizer app (https://automeris.io/WebPlotDigitizer) [33]. An a priori set random effects-model was used to pool individual study results. The DerSimonian and Laird inversed variance method was applied to calculate weight factors [34]. Studies were examined for clinical homogeneity, based on outcome, intervention and time of follow-up. To test the null hypothesis of no between studies heterogeneity a chi-square test with degrees of freedom and its corresponding p-value was applied. The degree of heterogeneity, i.e., the part of the total observed heterogeneity which can be explained by the true between studies heterogeneity, was expressed by Higgins’ *I*^2^. Higgins’ *I*^2^ benchmarking was used for interpretation of the calculated *I*^2^ values: around 25%: low heterogeneity; around 50%: moderate heterogeneity; around or above 75%: high heterogeneity [35]. The overall weighted, pooled estimate was expressed as an OR for re-injury prevalence or MD for re-injury incidence, with its corresponding 95% confidence interval. A subgroup meta-analysis was performed based on time of follow-up: ≤ 6 months of follow-up and 7–12 months of follow-up. Sensitivity analysis was performed to determine if quality of the exercise programs had an influence on pooled effect sizes. A fixed effect meta-regression was used to examine the effect of total rehabilitation time on re-injury risk. The association between the covariate (total rehabilitation time) and the primary outcome (log OR) was quantified using unstandardized regression coefficient (B), 95% CI and the corresponding p-value. Small study effects were examined using a funnel plot (standard error by log OR) and a classic fail-safe N test used to determine the number of statistically not significant studies it would need to bring the overall weighted mean effect size to statistically not significant [36]. Data extracted from individual studies regarding clinical and patient-reported outcomes were recalculated and expressed as between-group mean differences and effect sizes with 95%CI’s. For all statistical analyses level of error was set at 5% (p < .05).

## Results

### Search results and study flow

**Fig 1:**
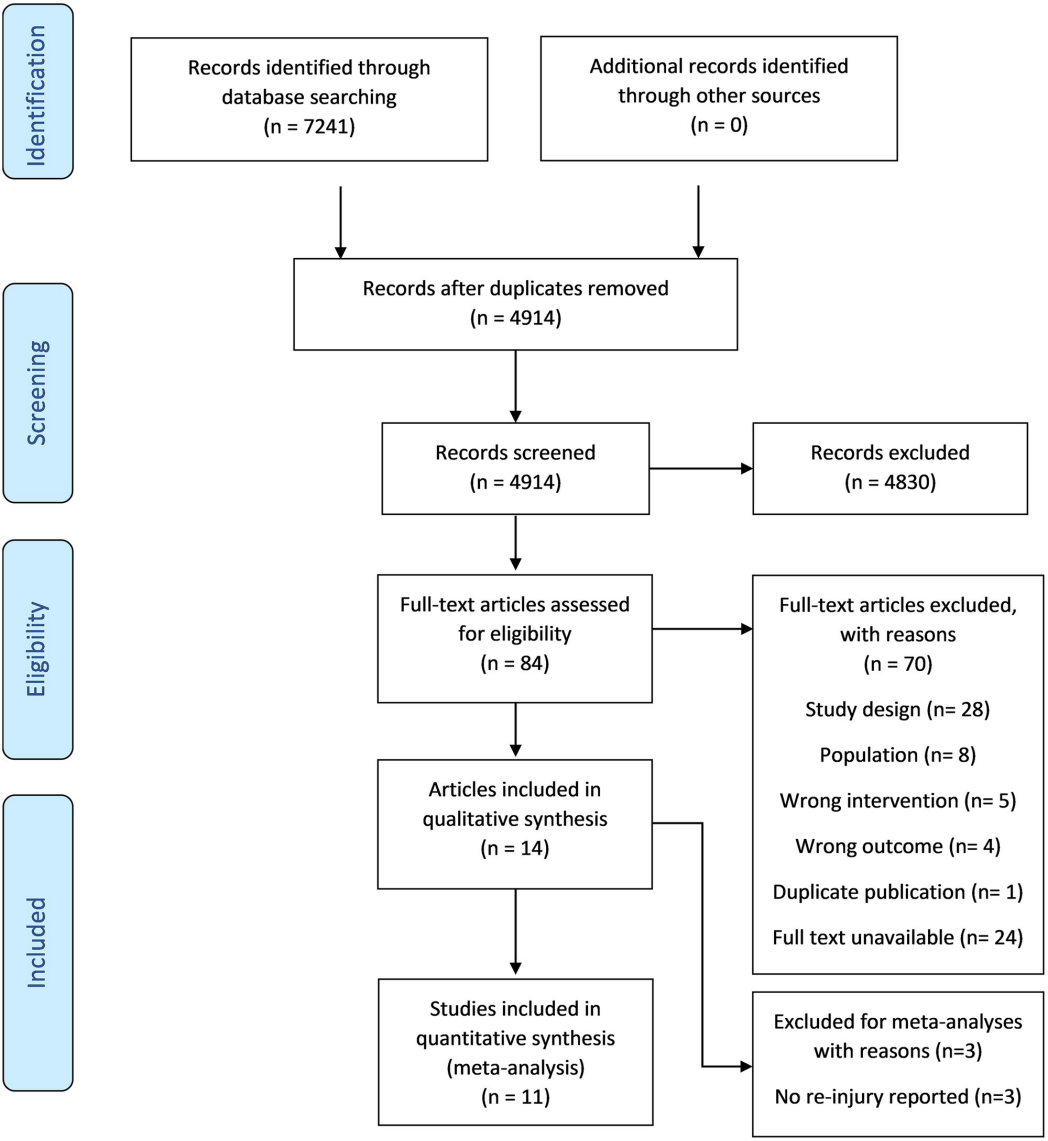
PRISMA flow diagram of the review process.

The literature search yielded a total of 7241 articles. After the removal of duplicates and both screening phases, fourteen eligible studies [37–50] were included for qualitative synthesis, of which eleven [37–42, 44, 46, 48–50] were included in meta-analysis. Full details of the review process are shown in the PRISMA flow diagram (Fig 1). A reference list containing studies which appeared to meet the eligibility criteria, but were excluded in the last phase of screening is provided in S2 File.

### Study characteristics

Table 1 summarizes the study characteristics. A total of 14 studies were included [37–50], representing a total of 2184 participants (52% male; 48% female). Study size varied from n = 20 to n = 522. Six [39, 43–46, 50] of the 14 studies failed to mention an a priori sample size calculation. The time of follow-up ranged from 1.5 months to 12 months. Participants were recruited from emergency departments, clinical practices, or from general practitioners, with time since injury at recruitment varying from day of injury to 11 weeks post sprain. In two studies, participants were recruited based on self-reported ankle sprains [42, 48], whereas all other studies used clinical assessments. Three studies specified a history of ankle sprain within the previous year or two years as a criterium for exclusion [40, 47, 49], and only two specified ankle syndesmosis injury as a criterion for exclusion [44, 45]. Six studies [39, 43, 46, 48–50] did not report on previous ankle sprain; in the others, this ranged from 42.5% [37] to 71% [42]. Exercise programs comprised postural balance exercises in all but 2 studies [37, 43]. The majority of studies used rehabilitation content which was either exclusively [46, 47, 50] or primarily based on postural balance training [39, 41, 42, 44, 45, 48, 49]. Nine [38–42, 44, 45, 48, 49] of the 12 studies used single-leg stance exercises, with applicable variations.

**Table 1:** Study characteristics.

Study	Participants A	Recruitment site (country)	Time since injury at recruitment	Diagnosis (n)	n. Previous ankle injury (%)	% re-injury: UC vs EB	Follow-up
**Bleakley** [37]	n = 101; 51–50	ED, sports injury clinic (Northern Ireland)	<7 days	Grade 1 (29)	42.5	4 vs 4	4 months
	69–32 (68–32%)			Grade 2 (73)			
	26y, SD: 8y						
**Brison** [38]	n = 503; 250–254	ED (Canada)	<72 hours	Grade 1 (149)	59.4	8.4 vs 7.5	6 months
	223–280 (44–56%)			Grade 2 (354)			
	30y, SD: 13y						
**Holme** [39]	n = 71; 42–29	ED (Denmark)	Day of injury	Grade 1 (21)	Not stated	29 vs 7	12 months
	44–27 (62–38%)			Grade 2 (38)			
	26y, SD: 4y			Grade 3 (12)			
**Hultman** [40]	n = 65; 32–33	ED (Sweden)	Day of injury	Ankle sprain	44.6	6 vs 3	3 months
	35–30 (54–46%)						
	35y, SD: 14y						
**Hupperets** [41]	n = 522; 256–266	ED, general practice, physical therapy offices (Netherlands)	<2 months	LAS	^47^	34 vs 30	12 months
	274–248 (52–48%)						
	28 y, SD: 12 y						
**Janssen** [42]*	n = 340; 113-107-120	Sport federations (Netherlands)	<2 months	Grade 1 (68)	71	15 vs 27 vs 19	12 months
	157–183 (46–54%)			Grade 2/3 (272			
	34 y, SD: 13 y						
**Kachanathu** [43]	n = 40; 20–20	University hospitals (Saudi Arabia)	Not stated	Grade 2 LAS	Not stated	Not stated	No follow-up
	24–16 (60–40%)						
	21.8 y, SD: 2.9 y						
**Lazarou** [44, 45]	n = 20; 10–10	Rehabilitation centre (Greece)	≥ 11 weeks	Ankle sprain	50	20 vs 0	12 months
	6–14 (30–70%)						
	22 y, SD: 3 y						
**Mohammadi** [46]*	n = 60; 20-20-20	Sports club (Iran)	Not stated	Ankle inversion sprain	Not stated	40 vs 5 vs 20	10 months
	60–0 (100–0%)						
	24.6 y, SD: 2.63 y						
**Punt** [47]*	n = 90; 30-30-30	ED (Switzerland)	4 weeks	Grade 1 (55)	60	0.03**	1.5 months
	51–39 (64–36%)			Grade 2 (35)			
	32.7 y, SD: 11 y						
**Van Reijen** [48]	n = 220; 110–110	Clinical practice, advertising (Netherlands)	<2 months	Grade 1 (91)	Not stated	63 vs 64	12 months
	110–110 (50–50%)			Grade 2 (64)			
	37.9 y, SD: 13.4 y			Grade 3 (18)			
**Van Rijn** [49]	n = 102; 53–49	General practitioner, ED (Netherlands)	<1 week	Grade 1 (43)	Not stated	31 vs 27	12 months
	59–43 (58–42%)			Grade 2 (42)			
	37 y, SD: 11.9 y			Grade 3 (4)			
**Wester** [50]	n = 48; 24–24	ED (Denmark)	Day of injury	Primary LAS; grade 2	Not stated	54 vs 25	8 months
	29–19 (60–40%)						
	25 y, SD: 7 y						

^A^ Participants: total amount; amount usual care—exercise-based rehabilitation; male-female (%); mean age, SD.

Abbreviations: n = amount; SD = Standard Deviation; UC = Usual care; EB: Exercise-based rehabilitation; SD: Standard deviation; ED = Emergency Department, LAS = lateral ankle sprain, ROM = range of motion.

*These studies included a 2-arm comparison: usual care—exercise-based intervention 1—Exercise-based intervention 2.

**No distinction was made.

### Risk of bias assessment

Table 2 shows the scores assigned to each study by the Risk of Bias (ROB) II Cochrane tool [32]. All studies showed low risk of bias for missing outcome data. Only one study [40] was judged to have some concerns regarding deviations from the intended interventions and one other study [43] was also deemed to have some concerns regarding measurement of the outcomes. Six studies [39, 40, 43, 46, 47, 50] did not pre-register their protocol or publish their methods, resulting in a judgement of “some concerns” for the category of selection of the reported results. Also six studies [39, 40, 43, 46, 48, 50] did not apply adequate randomisation, blinding participants and therapists, baseline comparability or allocation concealment, and where thus determined as “high risk” for the category of randomisation process.

**Table 2:** Risk of bias—ROB II Cochrane tool.

Study	1	2	3	4	5
**Bleakley** [36]	LR	LR	LR	LR	LR
**Brison** [37]	LR	LR	LR	LR	LR
**Holme** [38]	HR	LR	LR	LR	Q
**Hultman** [39]	HR	Q	LR	LR	Q
**Hupperets** [40]	LR	LR	LR	LR	LR
**Janssen** [41]	LR	LR	LR	LR	LR
**Kachanathu** [42]	HR	LR	LR	Q	Q
**Lazarou** [43, 44]	LR	LR	LR	LR	LR
**Mohammadi** [45]	HR	LR	LR	LR	Q
**Punt** [46]	LR	LR	LR	LR	Q
**Van Reijen** [47]	HR	LR	LR	LR	LR
**Van Rijn** [48]	LR	LR	LR	LR	LR
**Wester** [49]	HR	LR	LR	LR	Q

1. Randomisation process; 2. Deviations from the intended interventions; 3. Missing outcome data; 4. Measurement of the outcome; 5. Selection of the reported results

Abbreviations: LR = Low risk; Q = questionable/ Some concerns; HR = High risk.

### Therapeutic quality of exercise programs assessment

Each i-CONTENT criterion, and respective scores are stated in Table 3. There was a low risk of bias for patient selection, safety and exercise adherence. In all cases the type of exercise was deemed suitable and where applicable, a qualified supervisor was employed. The type and timing of outcome assessment was most inconsistent, with three of the included intervention programmes [43, 46] scoring high risk of bias and one [39] judged as probably not done due to insufficient information.

**Table 3:** Therapeutic quality of exercise program (i-CONTENT tool).

Study	1	2	3	4	5	6	7
**Bleakley** [37]	LR	LR	LR	NA	LR	LR	LR
**Brison** [38]	LR	PD	LR	LR	LR	LR	LR
**Holme** [39]	LR	PD	LR	PD	PnD	LR	LR
**Hultman** [40]	LR	LR	LR	LR	LR	LR	LR
**Hupperets** [41]	LR	LR	LR	NA	LR	LR	LR
**Janssen** [42]	LR	LR	LR	NA	LR	LR	LR
**Kachanathu** [43]	LR	PD	LR	LR	HR	LR	LR
**Lazarou** [44, 45]	LR	LR	LR	LR	LR	LR	LR
**Mohammadi** [46]—**proprioception**	LR	PD	LR	NA	HR	LR	LR
**Mohammadi** [46]—Strength*	LR	HR	LR	NA	HR	LR	LR
**Punt** [47]	LR	LR	LR	NA	LR	LR	LR
**Van Reijen** [48]	LR	LR	LR	NA	LR	LR	LR
**Van Rijn** [49]	LR	LR	LR	LR	LR	LR	LR
**Wester** [50]	LR	LR	LR	NA	LR	LR	LR

1. Patient selection; 2. Dosage of exercise program; 3. Type of the exercise program; 4. Qualified supervisor; 5. Type and timing of outcome assessment; 6. Safety of exercise program; 7. Adherence to the exercise program

Abbreviations: LR = “low risk” of ineffectiveness; HR = “High risk” of ineffectiveness; PD = Probably done; PnD = Probably not done; NA = Not applicable

* This study compared different types of exercise based intervention to usual care.

### Effect of interventions

Eleven of the included studies [37–39, 41, 42, 44–48, 50] compared no intervention or usual care to an exercise-based intervention. Four studies [40, 43, 47, 48] compared different exercise-based intervention modalities.

## Usual care vs exercise-based intervention

### Re-injury

**Fig 2:**
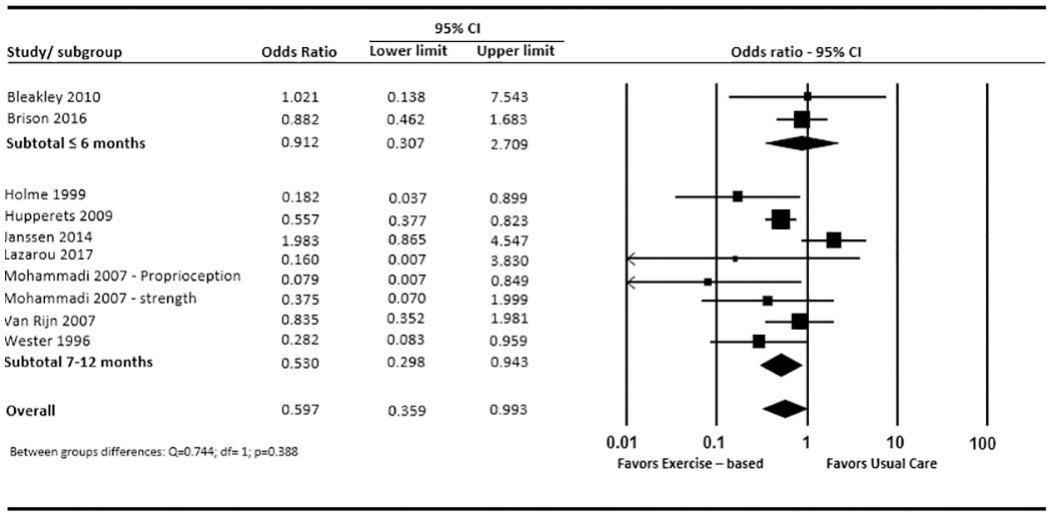
Forest plot (re-injury prevalence: Usual care vs exercise-based intervention).

In Fig 2, pooled data from nine studies [37–39, 41, 42, 44, 46, 49, 50], comprising ten intervention comparisons, shows a reduction in re-injury in favour of the exercise-based intervention group (OR: 0.61; 95%CI: 0.38 to 0.98). Subgroup analyses based on follow up time found statistically non-significant between group difference at ≤ 6 months in favour of the exercise-based intervention group (OR: 0.90; 95%CI: 0.31 to 2.71), while effect at 7–12 month follow up was statistically significant, again in favour of the exercise-based intervention group (OR: 0.53; 95%CI: 0.30 to 0.94). Sensitivity analysis excluding one study [46] which contained two intervention programs judged with a “high risk” of ineffectiveness [46], displayed that re-injury reductions in favour of the exercise-based rehabilitation group lost statistical significance, for both overall (OR: 0.68; 95%CI: 0.40 to 1.15) and subgroup analysis at 7–12 months follow-up (OR: 0.61; 95%CI: 0.33 to 1.13).

**Fig 3:**
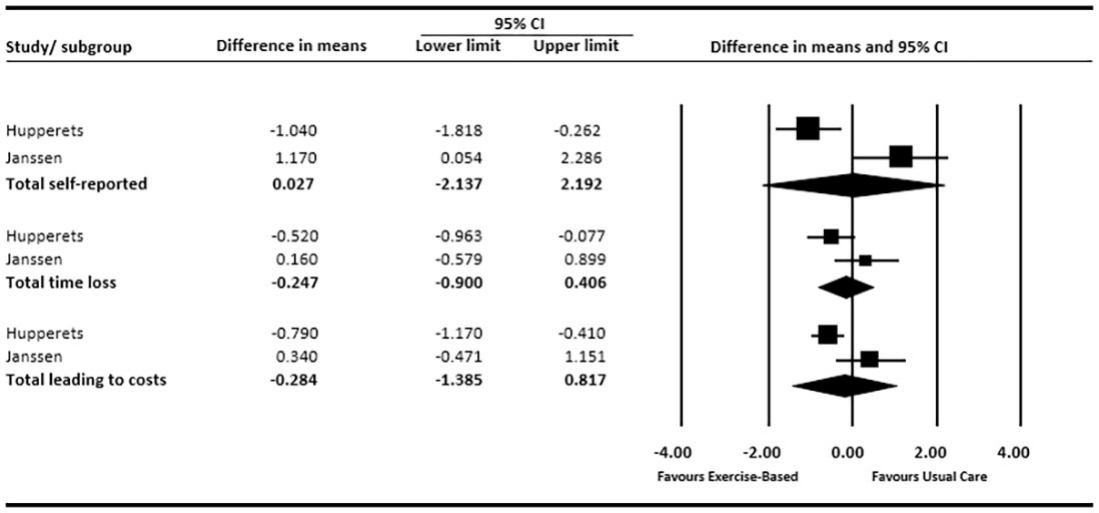
Forest plot (reinjury incidence: Usual care vs exercise-based intervention).

Two studies [41, 42] reported re-injury incidence rates, documented per 1000 hours of sports exposure. Pooled data in Fig 3, shows inconsistent effects across the two studies. The pooled effects were statistically non-significant for incidence of self-reported re-injury (MD: 0.027; 95%CI: -2.14 to 2.19), time-loss re-injury (MD: -0.25; 95%CI: -0.90 to 0.41) and costs (MD: -0.28; 95%CI: -1.39 to 0.82).

### Training volume

The majority of the included studies [38, 41–43, 46–49], reported a rehabilitation duration of 30 minutes per session with all other studies using durations between 10 and 60 minutes [37, 39, 40, 44, 45, 50]. The number of rehabilitation sessions ranged from 5 to 84 sessions, and the duration of rehabilitation ranged from 1 to 12 weeks. Total rehabilitation time was estimated for two studies [40, 43], and one study [46] failed to provide any information. In the remaining studies, total rehabilitation time ranged from 3.5 hours to 21 hours, with a median of 12 hours.

A fixed-method meta-regression analysis with pooled total rehabilitation time data from studies comparing usual care or no intervention to an exercise-based intervention showed no association between odds of re-injury and increasing training volume (r = -0.00086; SD: 0.00057; 95%CI: -0.00197 to 0.00025).

### Patient-reported outcomes

**Fig 4:**
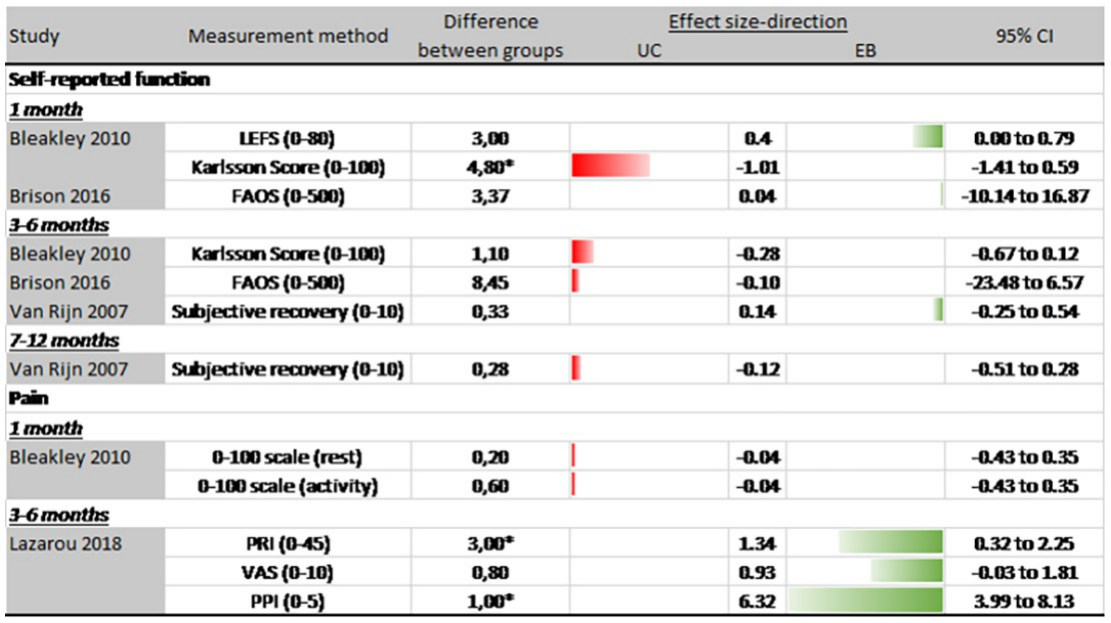
Patient-reported outcomes (usual care vs exercise-based rehabilitation). Abbreviations: UC = usual care; EB = exercise-based rehabilitation; 95% CI = 95% confidence interval; LEFS = Lower extremity function score; FAOS = Foot and ankle outcome score; PRI: Pain rating index; VAS = Visual analogue scale; PPI = Present pain index. * Significant difference (p < .05).

Fig 4 presents measurement methods, between-group mean differences and effect sizes with 95%CI’s for all patient-reported outcomes of studies comparing usual care to exercise-based rehabilitation at 1 month, 3 to 6 months and 7 to 9 months of follow up.

#### Perceived instability

Two [49, 50] studies assessing perceived instability favoured exercise-based intervention, but only the findings of Wester et al. [50] were statistically significant (p <0.01).

#### Self-reported function

Three studies [37, 38, 49] recorded self-reported function. Self-reported function results reported by Bleakley et al. [37] were consistently better for the usual care group at 1 month (ES: -1.01, 95%CI: -1.41 to 0.59) and 3–6 months of follow-up (ES: -0.28, 95%CI: -0.67 to 0.12), although only significant 1 month post injury. Results of Brison et al. [38] and Van Rijn et al. [49] were conflicting between different time points.

#### Pain

Three studies [37, 45, 50] reported pain outcomes at 1 month, 3–6 months or 7–12 months of follow up. Only Lazarou et al. [45] found significant differences between groups on pain outcomes. These results were in favour of the exercise-based intervention group after 3 to 6 months of follow-up for the pain rating index (ES: 1.34, 95%CI: 0.32 to 2.25) and present pain index (ES: 6.32, 95%CI: 3.99 to 8.13).

### Clinical outcomes

**Fig 5:**
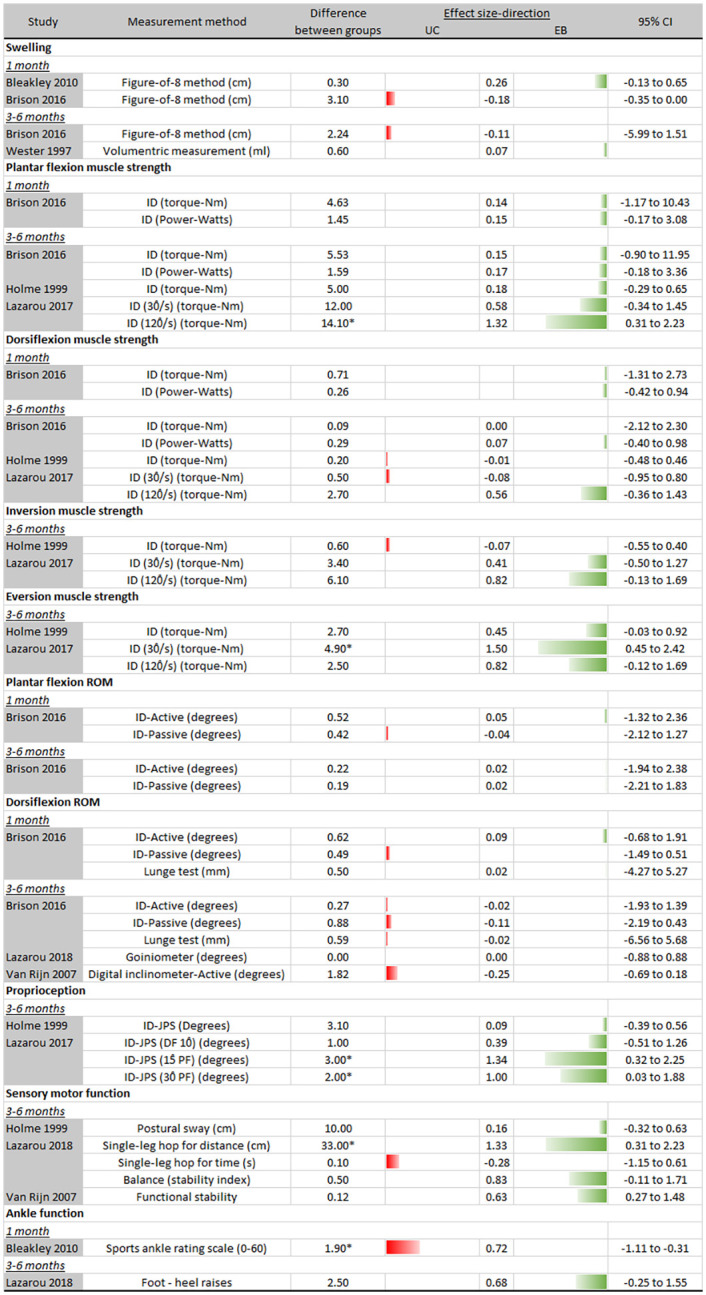
Clinical outcomes (usual care vs exercise-based rehabilitation). Abbreviations: UC = usual care; EB = exercise-based rehabilitation; 95% CI = 95% confidence interval; ID = Isokinetic dynamometer; JPS = Joint position sense; ROM = Range of motion. * Significant difference (p < .05).

Fig 5 depicts measurement methods, mean between-group differences and effect sizes with 95% CI of all clinical outcomes of studies comparing usual care to exercise-based rehabilitation at 1 month, 3 to 6 months and 7 to 9 months of follow up.

#### Swelling

Swelling was assessed in three studies [37, 38, 50], with two studies [37, 38] using the same method. Yet, results for this outcome were conflicting.

#### Muscle strength

Three studies [38, 39, 44] assessed muscle strength. Results of Brison et al. [38] and Holme et al. [39] were inconsistent, while Lazarou et al. [44] displayed significant effect sizes in favour of the exercise-based group for plantarflexion torque at 120°/s (ES: 1.32; 95%CI: 0.31 to 2.23) and eversion torque at 30°/s (ES: 1.50; 95%CI: 0.45 to 2.42) at 3–6 months of follow-up.

### Range of motion

Dorsiflexion range of motion (ROM) was evaluated by Brison et al. [38], Lazarou et al. [45] and Van Rijn et al. [49]. Results were inconsistent for both plantarflexion and dorsiflexion ROM at either 1 month and 3–6 months of follow up.

#### Proprioception

Two studies [39, 44] reported proprioception. Both studies found results favouring the exercise-based intervention group; however the largest effects were for plantarflexion joint position sense at 15°(ES: 1.34; 95%CI: 0.32 to 22.25) and 30°(ES: 1.00; 95%CI: 0.03 to 1.88) [44].

#### Sensory motor control

Three studies [39, 45, 49] assessed different components of sensorimotor control (postural sway, balance, single leg hop for distance, single leg hop for time, functional stability) at 3–6 months follow up. Effects were in favour of the exercise-based intervention group, except for balance performance. The only significant between group difference was for single-leg hop for distance (ES: 1.33; 95%CI: 0.31 to 2.23) [45].

#### Ankle function

Bleakley et al. [37] assessed ankle function at 1 month follow up using the sports ankle rating scale, reporting statistically significantly effects in favour of usual care (ES: -0.72; 95%CI: -1.11 to -0.31). Lazarou et al. [45] assessed ankle function 3–6 months post ankle sprain and found results in favour of the exercise-based intervention group, although statistically not significant (ES: 0.68; 95%CI: -0.25 to 1.55).

## Comparison between exercise-based interventions

**Fig 6:**
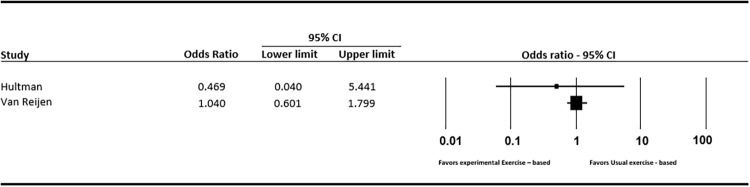
Forest plot (re-injury prevalence: Usual exercise-based vs experimental exercise-based).

Hultman et al. [40] compared supervised exercise-based intervention based on either two or four visits from a physiotherapist. Patients receiving four visits recorded a statistically non-significant reduction in re-injury (OR: 0.47; 95%CI: 0.04 to 5.44) (Fig 6). The group receiving four visits did have statistically better self-reported function (p<0.05), and lower pain (p = 0.025) at 3–6 months (Fig 8). There were no differences between groups for ankle plantarflexion and dorsiflexion ROM (p >.05), functional ankle stability (p = 0.86) and ankle function (p = 0.54).

**Fig 7:**
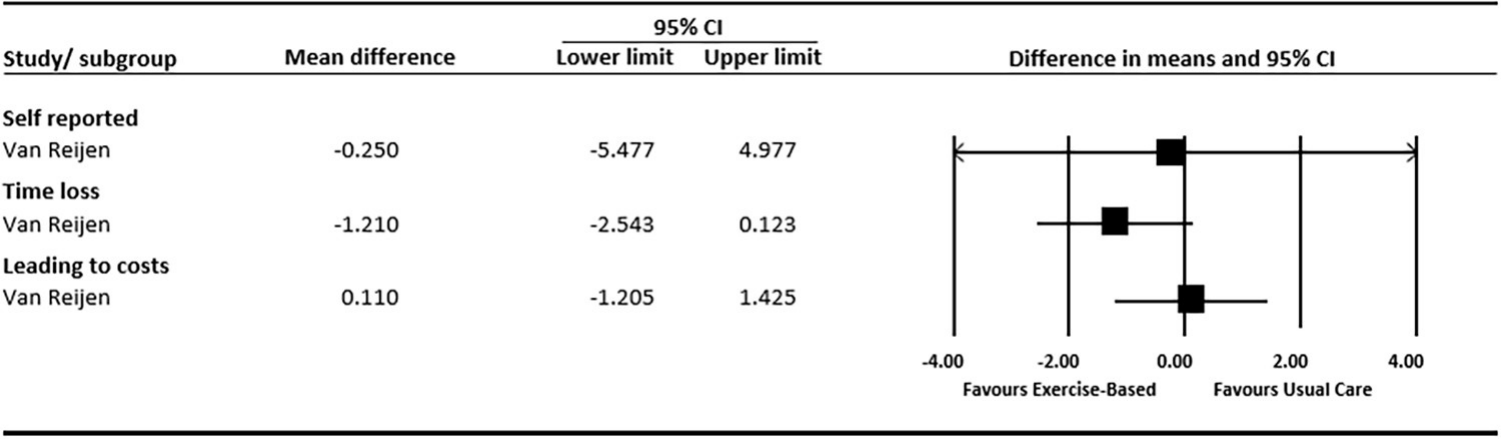
Forest plot (re-injury incidence: Usual exercise-based vs experimental exercise-based).

**Fig 8:**
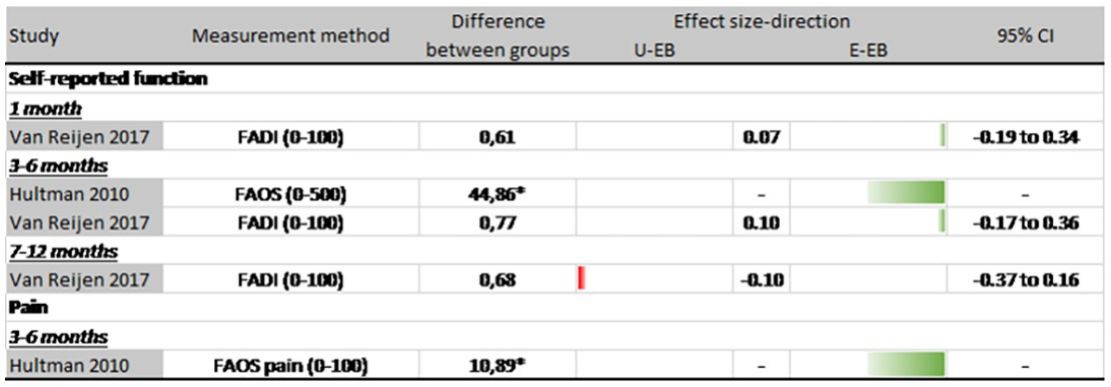
Patient-reported outcomes (usual exercise-based vs experimental exercise-based). Abbreviations: U-EB = Usual exercise-based rehabilitation; E-EB = Experimental exercise-based rehabilitation; 95% CI = 95% confidence interval; FADI = Foot and ankle disability index; FOAS = Foot and ankle outcome score. * Significant difference (p < .05).

Van Reijen et al. [48] compared an exercise-based rehabilitation program delivered via a phone application, to the same program provided in printed booklet. There was a small reduction in re-injury in favour of the booklet group (OR: 1.04; 95%CI: 0.60 to 1.80) but this was not statistically significant (Fig 6). Effects for self-reported re-injury incidence rates, presented as re-injuries per 1000 hours of sports exposure, favoured the phone application group, but again these were not statistically significant (MD: -0.25; 95%CI: -5.48 to 4.98). There were similar patterns for time-loss injuries (MD: -1.21; 95%CI: -2.54 to 0.12) (Fig 7). Self-reported function evaluated at 1 month, 3–6 months and 7–12 months follow up, also shows conflicting results (Fig 8).

## Discussion

### Summary of findings

Following a published decision framework [51], it was timely to update a previous systematic review [7]. The review doubles the number of included trials, with aggregate data from 2184 participants (48% female) across 14 RCTs. We found further evidence that exercise-based rehabilitation reduces the risk of recurrent ankle sprain vs minimal usual care. We used i-CONTENT to formally assess the therapeutic quality of the exercise interventions; and a sensitivity analysis found reduced effect magnitude (for re-injury risk), when lower quality interventions were removed from the meta-analysis. The content and volume of exercise interventions continues to vary, and using a planned meta-regression, there was a weak and non-significant trend that higher training volumes were most effective. Although, we extracted all available data on clinical and patient-reported outcomes, results were generally conflicting at most follow up time points.

### Re-injury

Pooled data from nine studies, found statistically significant reductions in re-injury in favour of the exercise-based rehabilitation group. The prevalence of recurrent ankle sprains was 22% in the usual care group, which aligns with previous prospective research [52]. By comparison, pooled prevalence of re-injury in the exercise group was 16%, equating to an absolute risk reduction of 6%. This effect magnitude is clinically important, and these findings must be disseminated to healthcare professionals, athletes and the general public. Research in the United States (US) shows ankle sprains to have the highest recurrence rates out of any musculoskeletal injury [53], yet many of the practitioners treating these injuries have a moderate understanding of their epidemiology and often limit management to rest-ice-compression-elevation [54]. Furthermore, the public perception, that ankle sprains are innocuous, remains widespread, and it is estimated that less than 50% of individuals who incur an ankle sprain, consult a medical professional [55].

The magnitude of re-injury risk reduction varied across the present set of studies. Four studies [39, 41, 46, 50] showed statistically significant reductions in odds of sustaining a re-injury in the exercise-based intervention group. Whereas others showed no difference between groups [37], or non-significant trends in favour of the usual care group [42] or the exercise-based rehabilitation group [38, 44, 46, 49]. Treatment effect may be moderated by many clinical factors, such as injury history, severity and method of diagnosis. In the current review, only two studies [44, 45] specified involvement of the ankle syndesmosis as a criterion for exclusion. It is estimated that 1 in 5 ankle sprains have syndesmotic involvement [56]; these injuries are associated with prolonged recovery [56] and therefore require a more conservative management strategy.

Only two studies compared different types of exercise-based interventions, but data pooling was not undertaken due to heterogeneity. Subsequently, no conclusion can be drawn regarding different types of exercise-based interventions. There was also some variation across studies, regarding the therapeutic quality of their exercise interventions based on the i-CONTENT tool. It was interesting that the study employing exercise interventions at highest risk of ineffectiveness (based on type and timing of outcomes, and their exercise dosage) [46], also reported the largest effect magnitudes. Again, we must acknowledge that there are many factors influencing effect estimate magnitude and direction. In particular, this study [46] also showed suboptimal methodological study quality [32], and failed to incorporate adequate allocation concealment, blinding of subjects, therapists and assessors, and intention-to-treat analysis. These limitations can yield wide CI’s and thus over estimate treatment effects [57].

### Exercise-based intervention

A pre-planned meta-regression showed a small decrease in odds of re-injury with increased training volume, although this was not statistically significant. We also found preliminary evidence from a single study [40] that four supervised training sessions is superior to two supervised sessions. We calculated training volume based on frequency, duration and timing, but a limitation was that no information regarding intensity was considered [58]. Training intensity is a key variable influencing training volume [59], but this has not yet been considered in the ankle literature. This may explain why, in contrast to other common lower limb injuries [27], we did not find a significant association between training volume and odds of re-injury. Consensus regarding optimal training volume and the effect of training volume on recurrent ankle sprains is still lacking in current literature. Moreover, consistency in intervention duration, number of rehabilitation sessions and time per rehabilitation session is warranted for further studies comparing ankle sprain rehabilitation.

### Clinical and patient-reported outcomes

Although our results displayed overall re-injury reductions, effects of rehabilitation on other important clinical outcome measures and patient-reported outcome measures are inconsistent. Others have shown that the course of recovery from an acute ankle sprain varies significantly, across clinical constructs (e.g., pain, subjective instability) [60, 61]. It is therefore important that this is reflected in the timing of outcome assessments.

The fifth criterium of the i-CONTENT tool [29], type and timing of outcome assessments, also displayed inconsistent results. This may also explain why our findings regarding re-injury reduction are conflicting with results of the secondary outcomes. Only Brison et al. [38] applied a criteria-based progression in their rehabilitation programme, addressing specific impairments, whereas all other studies applied a general intervention program independent of the progress made by the study participants. The rehabilitation progression of common musculoskeletal conditions such as Anterior Cruciate Ligament reconstruction (ACLR), calf muscle strain and hamstring injury are guided by evidence-based criteria. There are currently no criteria for rehabilitation progression after an acute ankle sprain [62].

### Strengths and limitations

Although we were able to double the amount of included studies of the previous review, only 9 studies were included for meta-analysis. Therefore, publication bias cannot be excluded [63]. Results of the ROB II assessment showed that only five [37, 38, 41, 42, 49] of these studies have low risk of bias [32]. Furthermore, after sensitivity analysis based on results of the i-CONTENT-tool both overall effect sizes and effect sizes at 7–12 months were not significant anymore. There was little consistency in measurement time points for clinical outcomes and patient-reported outcomes, and many studies provided insufficient data to calculate effect sizes; this limited the potential for data pooling. Where possible, we presented effect size estimates for individual studies, and we extracted outcome data at three clinically relevant time points: 1 months, 3–6 months and 7–12 months post ankle sprain. Guidelines for reporting outcomes in clinical trials are currently in development [64], which must be applied in future research. Similar to the previous review [7], we were unable to conclude the optimal exercise-based program based on content and volume, advocating future ankle sprain rehabilitation research to compile exercise-based rehabilitation programs in accordance with the Consensus on Exercise Reporting Template [65].

## Conclusion

Rehabilitation reduces the risk of recurrent ankle sprain by 40% compared to usual care or doing nothing. The effect of rehabilitation on other PROMs and clinical outcomes is conflicting. Exercise interventions were generally well reported but there was no evidence to suggest an optimal rehabilitation protocol or training volume. Better understanding of rehabilitation effect could be achieved through consistent measurement methods, with key focus on CAI prevalence and a minimal follow up of 12 months. Future research should examine which variables (duration, exercise content, intensity) have the greatest moderating effect on rehabilitation outcomes.

## Supporting information

S1 File MEDLINE search strategy. Search terms implemented for MEDLINE, modified as required and applied across other databases.
(DOCX)

S2 File. Reference list. Reference list of studies which might appear to meet inclusion criteria, but were excluded in second phase of screening (DOCX)

S3 File PRISMA 2020 checklist. (DOCX)

S4 File PRISMA 2020 abstract checklist. (DOCX)
