# Supplementary material for: Exercise-based rehabilitation reduces reinjury following acute lateral ankle sprain: A systematic review update with meta-analysis
Source: PLoS One. 2022 Feb 8;17(2):e0262023. doi: 10.1371/journal.pone.0262023 (PMC8824326; doi:10.1371/journal.pone.0262023)
Supplement: S1 File — Search terms implemented for MEDLINE, modified as required and applied across other databases. (DOCX) [file pone.0262023.s001.docx]

## S1 File: Search strategy

Databases: MEDLINE

The search of MEDLINE was optimized using the terms for the participants (ankle sprain) recommended by the Cochrane Collaboration. These search terms were modified for CINAHL, OVID EMBASE, Web of Science, Sports Discus, Cochrane Controlled Trials Register and the Physiotherapy Evidence Database.

1. Lateral ankle sprain
2. Ankle injuries
3. Ankle sprain
4. “Ankle AND sprain”
5. “lateral ligament, ankle”
6. “Chronic ankle instability”
7. Rehabilitation
8. Exercise therapy
9. Exercise
10. Neuromuscular
11. Physical therapy
12. Physiotherapy
13. Resistance training
14. Active therapy
15. OR/ 1-5
16. NOT/ 6
17. OR/ 7-14
18. 15 AND 16
19. Limit 15 to (randomized controlled trials and humans)
