# Supplementary material for: Exercise-based rehabilitation reduces reinjury following acute lateral ankle sprain: A systematic review update with meta-analysis
Source: PLoS One. 2022 Feb 8;17(2):e0262023. doi: 10.1371/journal.pone.0262023 (PMC8824326; doi:10.1371/journal.pone.0262023)
Supplement: S2 File. Reference list. Reference list of studies which might appear to meet inclusion criteria, but were excluded in second phase of screening — (DOCX) [file pone.0262023.s002.docx]

## S2 File: Reference list

Studies excluded which might appear to meet inclusion criteria, but were excluded in second phase of screening [1-74]

1. D. K, H.A. H, M.S.B. I. A12-weeks multimodal exercise training on ankle joint position sense in lateral ankle ligament injury of athletes. Research Journal of Pharmacy and Technology. 2018;11(9):4074-83.

2. Kemler H. Acute lateral ankle sprains: from functional treatment to prevention: Utrecht University; 2015.

3. H D. [All ankle traumas are not banal sprains...]. Revue medicale de la Suisse romande. 1999;119(10):839-45.

4. DR P, C J. Ankle sprains in young athletes. Part 2: how to manage and prevent. Contemporary Pediatrics. 2005;22(12):73-82.

5. Sempeles S. Balance Board Shows Some Effectiveness in Preventing Recurring Ankle Sprains. Sports Medicine Digest. 2004;26(11):121;6-7.

6. D P, CE H, KM R. Can the recurrence of ankle sprain be prevented by training ankle proprioception? J Orthop Sports Phys Ther. 2009;39(10):A23-A.

7. D A, R K, J B, C Z. The clinic. Ankle sprain return to running. Running & FitNews. 2005;23(5):9-10.

8. R S, S B, WO R. Clinical techniques. Rehabilitating ankle sprains. Physician & Sportsmedicine. 2002;30(8):48-50.

9. D S, Stasinopoulos D. Comparison of three preventive methods in order to reduce the incidence of ankle inversion sprains among female volleyball players. British journal of sports medicine. 2004;38(2):182-5.

10. M H, E V, W vM. Compliance with a 2-month home-based unsupervised proprioceptive training programme. British journal of sports medicine. 2008;42(6):523-.

11. R B, W M. [Conservative therapy of fibular ligament lesions]. Zeitschrift fur Unfallmedizin und Berufskrankheiten Revue de medecine des accidents et des maladies professionelles. 1981;74(3):161-3.

12. RS H. [Diagnosis, prevention and treatment of acute lateral ankle sprains]. Ugeskrift for laeger. 2019;181(8).

13. I. G, A.S. D, L. G, J. M, J. G, D. F. Does in-person physiotherapy intervention improve the functional recovery of children and adolescents with acute ankle sprains? A randomized clinical trial. Clin J Sport Med. 2017;27(3):e35.

14. Scherschligt BM, Evensen AE. Does supervised physical therapy after lateral ankle sprain reduce the risk of re-injury? Evidence-Based Practice. 2014;17(9):E-2.

15. Dinesha A, B AP. Effect of 3-week and 4-week wobble board exercise programme for improving the muscle onset latency and perceived stability in basketball players with recurrent ankle sprain. Indian Journal of Physiotherapy & Occupational Therapy. 2011;5(1):27-32.

16. TA M, JS K. The effect of a balance training program on the risk of ankle sprains in high school athletes. The American journal of sports medicine. 2006;34(7):1103-11.

17. E V, A vdB, J T, L B, R B, W vM. The effect of a proprioceptive balance board training program for the prevention of ankle sprains: a prospective controlled trial. United States2004 2004-9. 1385-93 p.

18. M M-E, N F, B D. The effect of balance training on rate of pain, muscle force, and balance performance in both feet of patients with ankle sprain. J Orthop Sports Phys Ther. 2009;39(10):A22-A.

19. T Y, Y T, T S. Effect of exercise therapy combining electrical therapy and balance training on functional instability resulting from ankle sprain-focus on stability of jump landing. Journal of physical therapy science. 2015;27(10):3069-71.

20. CR L, MR H, J G, KY K, J P, KK C. Effect of Neuromuscular Warm-up on Injuries in Female Soccer and Basketball Athletes in Urban Public High Schools: Cluster Randomized Controlled Trial. Archives of Pediatrics & Adolescent Medicine. 2011;165(11):1033-40.

21. H.M. L, S. O, J.W. K. Effect of plyometric versus ankle stability exercises on lower limb biomechanics in taekwondo demonstration athletes with functional ankle instability. International Journal of Environmental Research and Public Health. 2020;17(10):3665.

22. EH B, SP M, K B, T B, P A, M K. [The effect of supervised rehabilitation on ankle joint function and the risk of recurrence after acute ankle distortion]. Denmark2001 2001-6-4. 3223-6 p.

23. Faizullin I, Faizullina E. Effects of balance training on post-sprained ankle joint instability. International Journal of Risk & Safety in Medicine. 2015;27:S99-S101.

24. NS MS, MA U, S S. Effects of the standard physiotherapy programme on pain and isokinetic ankle strength in individuals with grade I ankle sprain. Journal of Taibah University Medical Sciences. 2018;13(6):576-81.

25. Cho B-K. Evidence-based Treatment of Acute Lateral Ankle Sprain. Journal of Korean Foot and Ankle Society. 2018;22(4):135-44.

26. JA H, TL S. Exercises for foot and ankle injuries. Consultant (00107069). 1997;37(8):2228-30.

27. Exercises that help an injured ankle. Patient Care. 1992;26(4):31-2.

28. JF C. Exercises to help you recover from a sprained ankle. Consultant (00107069). 1996;36(8):1644-6.

29. Grotle M, Hagen KB. Fonction de la cheville après entorse : amélioration à court terme par l'association exercices précoces, glace et contention intermittente...Effect of accelerated rehabilitation on function after ankle sprain: randomised controlled trial. Kinesitherapie Revue. 2014(155):16-.

30. D T. Functional rehab in ankle injuries. Rehab Management: The Interdisciplinary Journal of Rehabilitation. 1994;7(5):31-132.

31. G W. Functional rehabilitation. A protocol for management of the lateral ankle sprain. Rehab management. 1996;9(4):54-60.

32. M H. [The functional treatment of sprains of the external lateral ligament of the ankle]. Revue medicale de la Suisse romande. 1992;112(10):893-5.

33. Health tips. Strengthening ankles. Mayo Clinic health letter (English ed). 2003;21(1):3.

34. SF B, H P. Home-based physical therapy intervention with adherence-enhancing strategies versus clinic-based management for patients with ankle sprains. Physical therapy. 2007;87(9):1132-43.

35. SF B, H P. Home-based physiotherapy versus clinic-based physiotherapy rehabilitation for people with acute ankle sprains. New Zealand Journal of Physiotherapy. 2008;36(2):83-.

36. M VR, II V, V Z, W vM, EA V. The implementation effectiveness of the 'Strengthen your ankle' smartphone application for the prevention of ankle sprains: design of a randomized controlled trial. BMC musculoskeletal disorders. 2014;15:2.

37. MV S, WC dL. [Importance of proprioceptive training in sprained ankle rehabilitation]. Fisioterapia em Movimento. 2003;16(2):27-34.

38. T W, H B, A W, H Z, S R. Influence of a proprioceptive training on functional ankle stability in young speed skaters - a prospective randomised study. Journal of sports sciences. 2015;33(8):831-40.

39. Deußen S, Alfuth M. The influence of sensorimotor training modalities on balance, strength, joint function and foot sensitivity in recreational athletes with a history of ankle sprain: a randomized controlled pilot study...Second World Congress of Sports Physical Therapy, October 6-7 2017, Belfast. Phys Ther Sport. 2017;28:e25-e.

40. Information from your family doctor. How to care for your ankle sprain. American family physician. 2012;85(12):1.

41. Is home rehabilitation as effective as supervised PT after acute ankle sprain? Joint Letter. 2001;7(5):52-3.

42. RL B. 'It's only a sprained ankle'. American family physician. 1972;6(4):68-75.

43. CR T, AC K. Lower-Extremity Rehabilitation. Current sports medicine reports. 2018;17(12):405-6.

44. AA L. Management of sprained ankles. A double-blind study. England1981 1981-6. 935-6 p.

45. M C. Managing acute ankle sprains. Dynamic Chiropractic. 2008;26(18):44-.

46. JA C, PE M, A M, ML B, KJ C, K K, et al. Manual physical therapy and exercise versus supervised home exercise in the management of patients with inversion ankle sprain: a multicenter randomized clinical trial. United States2013 2013. 443-55 p.

47. D. K, H.A. H, M.S.B. I. Muscle strength and balance training in subjects with lateral ankle ligament injury of athletes. Research Journal of Pharmacy and Technology. 2018;11(9):3841-7.

48. C R, R I, R S, C C, S C. Noninvasive Interactive Neurostimulation Therapy for the Treatment of Low-Grade Lateral Ankle Sprain in the Professional Contact Sport Athlete Improves the Short-Term Recovery and Return to Sport: A Randomized Controlled Trial. The Journal of foot and ankle surgery : official publication of the American College of Foot and Ankle Surgeons. 2019;58(3):441-6.

49. Patient information. Caring for ankle sprains. Advance for nurse practitioners. 2001;9(4):47.

50. G T. Physiotherapy in the management of sports injuries. The Medical journal of Malaysia. 1979;33(3):277-8.

51. I. T. Physiotherapy plan does not benefit simple ankle sprains, study finds. BMJ (Online). 2016;355:i6159.

52. OBA O, LM P-D, CA E. Prevention of Ankle Sprain Injuries in Youth Soccer and Basketball: Effectiveness of a Neuromuscular Training Program and Examining Risk Factors. Clinical journal of sport medicine : official journal of the Canadian Academy of Sport Medicine. 2018;28(4):325-31.

53. V G, M vS, E V, J Z. The prevention of musculoskeletal injuries in volleyball: the systematic development of an intervention and its feasibility. Injury epidemiology. 2017;4(1):25.

54. D R, R B, F R, C M. Proprioceptive Training and Injury Prevention in a Professional Men's Basketball Team: A Six-Year Prospective Study2016 2016-2. 461-75 p.

55. R.M. BdM, L. BdM. Proprioceptive training for functional instability of the ankle. Phys Ther Sport. 2020;45:e6.

56. Recovering from an ankle sprain. Harvard women's health watch. 2003;10(8):2-3.

57. Recovering from an ankle sprain. Take it easy, but keep moving. Harvard women's health watch. 2007;14(6):4-6.

58. Malone T. Rehabilitation after the ankle ligament sprain. Deerfield Beach, Fla.: Transcripts for Tomorrow, Inc.; 1980 1980.

59. LD M. Rehabilitation exercises following inversion ankle sprains. Journal of the American Podiatric Medical Association. 1986;76(10):577-81.

60. JE D. Rehabilitation of ankle sprains. Clinics in sports medicine. 1989;8(4):877-91.

61. M. B, R. G, E. S, P. B. The rehabilitation of ankle sprains in sport. Actual role of the proprioceptive exercises. Chirurgia del Piede. 1991;15(2):163-7.

62. CA P. Rehabilitative exercises following ankle injuries. Orthopedics. 1990;13(7):723-5.

63. RM vR, JA vH, P vdW, BW K, SM B-Z. Some benefit from physiotherapy intervention in the subgroup of patients with severe ankle sprain as determined by the ankle function score: a randomised trial. The Australian journal of physiotherapy. 2009;55(2):107-13.

64. JN W. Specific injuries of sport. Physiotherapy. 1972;58(6):194-9.

65. SG P, KA M, SA B, SA B, MT C. Sports Injuries of the Foot and Ankle: How Do Treatments Differ From the General Patient Population. United States2017 2017-8. 329-32 p.

66. S N. Sprains of the lateral ankle ligaments. Norway1983 1983-2. 13-36 p.

67. D C, E L, L N. Star excursion balance training: effects on ankle functional stability after ankle sprain. Journal of the Medical Association of Thailand = Chotmaihet thangphaet. 2005;88:S90-4.

68. A. A, T. I, R. S. Therapy and recurrence prevention of the ankle sprain in elite soccer players. Gazz Med Ital Arch Sci Med. 2016;175(1):23-6.

69. JU W, SM J, KD N, L N. [Training on a wobble board following lateral ankle joint sprains]. Denmark1998 1998-1-26. 632-4 p.

70. S R, AB M. Treatment of inversion injuries of the ankle by early active management. Physiotherapy. 1983;69(10):355-6.

71. SD B. Treatment of the sprained ankle. The Journal of the American Osteopathic Association. 1980;79(11):680-92.

72. C. L. Unsupervised home-based proprioceptive exercises reduces the risk of secondary ankle sprain. Kinesitherapie. 2013;13(136):8-9.

73. TT Ø, MA R. Warm-up exercise prevents acute knee and ankle injuries in young handball players. The Australian journal of physiotherapy. 2005;51(2):131.

74. KM R, J R, CE H, S K. Wobbleboard training has no effect on balance and a selective effect on proprioception in recurrent ankle sprain. J Orthop Sports Phys Ther. 2009;39(10):A14-A.
